# Depression and physical activity in a sample of nigerian adolescents: levels, relationships and predictors

**DOI:** 10.1186/1753-2000-5-16

**Published:** 2011-05-14

**Authors:** Ade F Adeniyi, Nkechi C Okafor, Celia Y Adeniyi

**Affiliations:** 1 Department of Physiotherapy, College of Medicine, University of Ibadan, Ibadan, Nigeria; 2 Department of Psychiatry, University College Hospital, Ibadan, Nigeria

## Abstract

**Background:**

Physical inactivity is related to many morbidities but the evidence of its link with depression in adolescents needs further investigation in view of the existing conflicting reports.

**Methods:**

The data for this cross-sectional study were collected from 1,100 Nigerian adolescents aged 12-17 years. Depressive symptomatology and physical activity were assessed using the Children's Depression Inventory (CDI) and the Physical Activity Questionnaire-Adolescent version (PAQ-A) respectively. Independent t tests, Pearson's Moment Correlation and Multi-level logistic regression analyses for individual and school area influences were carried out on the data at p < 0.05.

**Results:**

The mean age of the participants was 15.20 ± 1.435 years. The prevalence of mild to moderate depression was 23.8%, definite depression was 5.7% and low physical activity was 53.8%. More severe depressive symptoms were linked with lower levels of physical activity (r = -0.82, p < 0.001) and moderate physical activity was linked with reduced risk of depressive symptoms (OR = 0.42, 95% CI = 0.29-0.71). The odds of having depressive symptoms were higher in older adolescents (OR = 2.16, 95% CI = 1.81-3.44) and in females (OR = 2.92, 95% CI = 1.82-3.54). Females had a higher risk of low physical activity than male adolescents (OR = 2.91, 95% CI = 1.51-4.26). Being in Senior Secondary class three was a significant predictor of depressive symptoms (OR = 3.4, 95% CI = 2.55-4.37) and low physical activity.

**Conclusions:**

A sizable burden of depression and low physical activity existed among the studied adolescents and these were linked to both individual and school factors. Future studies should examine the effects of physical activity among clinical samples of adolescents with depression.

## Introduction

There is currently widespread recognition of the immense burden that depression imposes on individuals, communities and health services throughout the world [1]. Depression, which is the most common form of emotional problems experienced during adolescence, can be characterized by feelings of sadness, anxiety, fear, guilt, anger, contempt and confused thinking [2]. It has been shown that most adults who experience recurrent episodes of depression had an initial depressive episode as teenagers [3,4], suggesting that adolescence is an important developmental period in which to intervene [4]. According to Dunn and Weintraub [5], successful treatÂ¬ment of teen depression is important not only in reducing the suffering, morbidÂ¬ity, and mortality resulting from the disorder but also in preventing the development of other adverse long-term psychosocial and health outcomes.

Regular participation in physical activity not only benefits adolescents by strengthening the muscles, improving bone mass, sustaining oxygen uptake, reducing risk of cardiovascular and other chronic diseases, but also helps to improve self-esteem, increase self-consciousness and reduce anxiety and stress [6]. Although service access and treatment coverage remain low, there is growing empirical evidence from low-income as well as high-income countries on the effectiveness and cost-effectiveness of a range of pharmacological and psychosocial interventions for treating and managing depression [1]. However, despite a dramatic increase in the number of intervention studies on major depressive disorders in adolescents in the past 15 years, the majority being clinical trials of medications and cogniÂ¬tive behavioural therapy, response rates have been modest and remission rates low [5]. On the other hand, findings have supported the protective effects of physical activity on depression for older adults and cross-sectional analyses have shown that an association exists between physical activity and depression even when adjustments were made for a relatively large number of potentially confounding variables [7]. It has also been shown that regular physical activity may improve a variety of physiological and psychological problems in depressive persons [8]. In spite of all these, not many experimental studies have been done to support this assumption for adolescent populations [5,8]. A preliminary step to such studies, especially in a developing country like Nigeria, is to establish the prevalence of depression and the extent of engagement in physical activity, and the relationship between these. In Norwegian adolescents, Sagatun et al [9] had reported that emotional symptoms at age 18-19 were inversely associated with physical activity at age 15-16 in both genders, while a study carried out in an East London community found that there was evidence for a cross-sectional association between physical activity and depressive symptoms for both boys and girls at baseline, with a decrease in the odds for depressive symptoms of about 8% for each additional hour of exercise undertaken per week [10].

As has been the case with the development of most other treatments of paediatric psychiatric disorders that are also common in adulthood, it is necessary to extrapo-late from adult studies of exercise treatÂ¬ment of depression when justifying the need for research about physical activity in adolescent populations [5]. According to Dunn and Weintraub [5], virtually all well-designed studies on depression have been conducted only in adult populations. In addition, prior studies did not examine the relationships between physical activity and depression in a large sample of adolescents from Nigeria, and data on level of depression and physical activity appear to be irresolute. At the moment, research in Western countries has revealed a link between depression and physical activity, yet these may not fully represent the situation in a developing nation like Nigeria. This may be because of disparities in knowledge, and attitude towards physical activity, socio-economic background, educational curricula and existing policies. The present study explores (1) the prevalence of depression and physical activity levels, (2) the relationship between depression and physical activity and (3) selected demographic factors that may be linked with depressive symptomatology and low physical activity among a sample of Nigerian adolescents.

## Methods

### Study design

This study was a cross-sectional survey of Nigerian adolescents from Ibadan North Local Government Area of Oyo State, South Western Nigeria.

### Participants

The data from this cross-sectional study were collected from urban dwelling secondary school adolescents aged 12-17 years. The Children's Depression Inventory (CDI) and the Physical Activity Questionnaire, Adolescent Version (PAQ-A) were administered on 1,100 secondary school students from a population of approximately 100,000 secondary school students in the Ibadan North Local Government Area of Oyo State. The sample size was estimated to produce a precision level of ±3% at 95% confidence level and a degree of variability of 0.5 [11].

The study used a stratified, two-stage sampling technique to select participants for the study to meet the sample size requirement. The first stage was the selection of schools from both the private and public secondary schools in the local government area. In Nigeria, private and public schools operate side by side at all levels of education ranging from primary to secondary to tertiary educational institutions. The private schools are owned by individuals and the management determines the welfare of the students and teachers. In the public schools, the administration is entirely by government. However, as much as possible, both groups of schools operate a similar curriculum. Except for special reasons, conducting studies in only one of the types of school would not give a true picture of the issue under investigation. The schools were however varied in their population; the government schools had more students than the private ones. In the first stage of the sampling, schools were selected randomly based on a probability proportional to the total number of private or public secondary schools. Eleven schools (six public and five private) were selected for inclusion in the study.

In the second stage, 100 students from each school were drawn at random from the list of students in the senior secondary classes one to three. This produced the total sample of 1,100 adolescents that were surveyed. The classes were made up of younger adolescents (less than 15 years) and older adolescents (15 years and above) within the age range of 13 to 17 years. Although the level of adolescence and class of study appear to be similar, they are however different. An older adolescent is normally expected to be found in a more senior class but this situation is not always true as there are situations when younger adolescents were found in the highest class of study and vice-versa. This explains why the two variables were treated separately in this study.

This study was approved by the Joint University of Ibadan and the University College Hospital Research Ethics Committee (Approval ID No: UI/EC/10/0064). Written informed assent was obtained from all participants as well as their parents. Approval was also obtained from the management of each of the schools for the study to be carried out in their respective schools.

### Data collection procedure

Prior to data collection, the students were formally informed of the purpose of the study in an assembly in the school hall, in their classrooms or any other convenient place. The students were also informed of their right to decline participation. Before administering the PAQ-A and the CDI questionnaires on the selected participants, they were pre-tested on five students from each of the selected schools (total of 55 students) to identify areas of potential difficulty in filling the forms. Participants were comfortable with all the questions on the CDI but had problems mainly with the PAQ-A questionnaire because some questions sought information on their participation in a number of sporting activities that were more or less alien to them. For instance, the students needed help in understanding activities like in-line skating, skateboarding, ice-skating and ice hockey/ringette. Because the questionnaire was adopted from a different environmental setting, it was necessary to allow for differences in comprehension due to situational, cultural or semantic factors. Subsequently the questionnaire was modified by removing the "strange" sporting activities and replacing them with more familiar local sporting activities such as ten-ten and lakanlaka (these are games played with one or more partners, respectively, and involve hopping/running and stretching of the legs). In addition to the information drawn from the PAQ-A and the CDI questionnaires, information was also obtained on some demographic characteristics of the participants. These included information about age, sex and class of study.

### Assessment of depression

Depression was assessed using the CDI developed by Maria Kovacs. The CDI was designed to measure self-rated, symptom oriented assessment of depressive symptoms for school age children and adolescents. Subscales in the CDI included negative mood, interpersonal problems, ineffectiveness, anhedonia (the inability to gain pleasure from normally pleasurable experiences) and negative self-esteem. It covers the consequences of depression as they relate to children and functioning in school and with peers [12]. A reliability coefficient of 0.86 was reported for the scale and found to be a valid measuring device when compared with other instruments [13]. For each of the 27 items, the participant has three possible answers; 0 indicating an absence of symptoms, 1 indicating mild symptoms, and 2 indicating definite symptoms. The total score ranged from 0 to 54, with higher scores representing more severe depressive symptomatology. Participants were classified according to cut-offs proposed by Kovacs [13], which minimise the risk of false positives, whereby a CDI score of 0 indicates no symptoms, scores 1-19 indicate 'mild to moderate' depressive symptoms and scores equal to or above 20 indicate 'definite caseness' [13-15]. This classification was applied since there was no specific cut-off point for CDI based on studies carried out on Nigerian adolescents. Rivera et al [15] argued that a lower cut-off point is only usually suggested for populations where high rates of depression are expected.

### Assessment of physical activity

The PAQ-A (a slightly modified version of the PAQ-C for children) is a self-administered, 7-day recall instrument. It was developed to assess general levels of physical activity for high school students approximately 13 to 19 years of age. It assesses frequency of participation in physical activities such as sports or activities that make participants sweat or make their legs feel tired, or games that make participants breathe hard, such as skipping, running, and climbing. The PAQ-A also sought information regarding physical activity during spare time, physical education period and lunchtime, as well as after school, in the evenings and on weekends. For example: "In the last 7 days, during your Physical Education classes, how often were you very active (playing hard, running, jumping throwing)?" Participants respond on a five-point Likert scale. A 'summary of physical activity score' is generated from the mean of 8 items, and ranges from 1-5, with higher scores indicating more frequent participation in physical activity [16]. Those with low physical activity level were those who scored between 1 to 1.9 on the PAQ-A instrument while moderate and high physical activity levels were recorded for those who scored between 2 to 3.9 and 4 to 5 respectively on the PAQ-A. In a study to establish the convergent validity of the PAQ-A, the instrument was found to be significantly correlated to all self-report measures (including activity rating, r = 0.73; Leisure Time Exercise Questionnaire, r = 0.57; and 7-day physical activity recall interview, r = 0.59) [17].

### Statistical Analyses

Statistical analyses were conducted using the SPSS Version 15.0 (Chicago, USA) and STATA version 10.0 (Texas, USA). Results are presented using frequencies and percentages. Independent t-test were used to compare the mean CDI and PAQ-A scores between private and public schools, between younger and older adolescents, and between male and female participants; while the Analysis of Variance (ANOVA) was used to compare the scores obtained for the three class levels from which the adolescents were recruited. Scheffe's post hoc analysis was used to indicate the areas of significance in the three class levels. Pearson's moment correlation was used to assess the relationship between the CDI and PAQ-A scores while a further coefficient of determination (r^2^) was calculated to reveal the amount of variability in the depression level that the physical activity of the participants may account for.

Multi-level logistic regression analyses with students nested within schools was conducted. This was done at two levels with individual influences being the first level and school influences being the second level. The individual level variables included age, sex and physical activity levels while school levels included class of study and type of school. Bivariate analysis was carried out for the variables at both levels controlling for age and sex. Variables that showed significant associations in the bivariate model were introduced in the multivariable models. Multivariable analysis was initially performed separately for individual and school levels. The influence of individual factors and school level factors on depression and low physical activity were separately assessed through different models. Level of significance was at p < 0.05.

## Results

### Demographic characteristics of participants

The demographic characteristics of the participants are shown in table 1. The sample was made up of 538 boys (48.9%) and 562 girls (51.1%) with an overall mean age of 15.20 ± 1.435 years. The 1,100 participants were recruited from the Senior Secondary (SS) classes of eleven secondary schools with 691 (62.8%) of them from the SS 2 class.

**Table 1:** Bio-data of the participants

	Public	Private	Total
**Gender**			
Male	295 (49.2%)	243 (48.6%)	538 (48.9%)
			
Female	305 (50.8%)	257 (51.4%)	562 (51.1%)
**Total**	**600 (54.5%)**	**500 (45.5%)**	**1,100 (100%)**
			
**Class of Respondents**			
SS1 (14.2 ± 1.2 years)	48 (8%)	299 (59.8%)	347 (31.6%)
SS2 (15.9 ± 1.8 years)	515 (85.8%)	176 (35%)	691 (62.8%)
SS3 (17.1 ± 1.6 years)	37 (6.2%)	25 (5%)	62 (5.6%)
	**600 (54.5%)**	**500 (45.5%)**	**1100 (100%)**
**Level of adolescence**			
Younger adolescent	426 (71%)	262 (52.4%)	688 (62.5%)
Older adolescent	174 (29%)	238 (47.6%)	412 (37.5%)
	**600 (54.5%)**	**500 (45.5%)**	**1100 (100%)**
			
Mean age (years)	15.87+ 1.277	14.40 +1.180	15.20+1.435

SS = Senior Secondary.

### Levels of depression and physical activity of the adolescents

As presented in table 2 a total of 776 (70.5%) of the students had no symptoms of depression (score of zero on the CDI), while 262 (23.8%) had mild to moderate symptoms (score between 1 and 19 on the CDI), and 62 (5.7%) had definite symptoms (score ≥ 20). The physical activity levels of the participants ranged from low to moderate to high with 592 (53.8%) having low physical activity level. A total of 427 (38.8%) participants had moderate physical activity level while 7.4% reported high physical activity.

**Table 2:** Levels of depression and physical activity of the adolescents


CDI	Symptoms of Depression		
	**Absent**	**Mild to moderate**	**Definite**
	(CDI = 0)	(CDI = 1-19)	(CDI > 20)
	776 (70.5%)	262 (23.8%)	62 (5.7%)
			
PAQ-A	*Physical activity level		
	**Low**	**Moderate**	**High**
	(PAQ-A = 1-1.9)	(PAQ-A = 2-3.9)	(PAQ-A = 4-5)
			
	592 (53.8%)	427 (38.8%)	81 (7.4%)
			

*Physical activity score 1 = low, 5 = high [16]. Ratings between >1 and <5 are presented for ease of categorisation.

### The CDI and PAQ-A scores of the adolescents

The mean depression score (table 3) measured by the CDI for the adolescents in the private secondary schools was 14.2 ± 3.5 and this was significantly higher than the mean CDI score of 11.6 ± 4.1 for the adolescents in the public schools (t = 11.18, p < 0.0001). The mean score of physical activity measured by the PAQ-A for adolescents in the private schools (1.6 ± 0.3) was significantly lower (t = 35.69, p < 0.0001) than that of the public schools. The males presented with significantly (t = 14.13, p < 0.00001) lower depression scores and significantly (t = 71.83, p < 0.0001) higher physical activity scores than the females. In terms of classification of the participants based on their age, those classified as older adolescents (age 15 years and over) had significantly lower mean physical activity scores and significantly higher depression scores than the younger adolescents (younger than age 15 years). The mean scores for physical activity were fairly stable (about 2.4) between the two lower classes of the Senior Secondary schools (SS1 and SS2) but dropped significantly (F = 80.23, p = 0.003) by SS3 which was the most senior class in the senior secondary category. Post hoc analysis showed a significant difference in mean physical activity score between the SS3 class and each of the two lower classes.

**Table 3:** PAQ-A and CDI scores by type of school, sex, level of adolescence and class of study

	Mean CDI	Mean PAQ-A
**Type of school**		
Private (n = 500)	14.2 ±3.5	1.6 ± 0.3
Public (n = 600)	11. 6 ± 4.1	2.8 ± 0.7
		
t	11.1847	35.6959
p value	<0.0001	<0.0001
		
**Sex**		
Males (n = 538)	8.8 ± 3.9	3.6 ± 0.6
Females (n = 562)	13.5 ± 6.7	1.4 ± 0.4
		
t	14.1386	71.8344
p value	<0.0001	<0.0001
**Level of adolescence**		
Younger adolescents (n = 688)	9.3 ± 3. 2	3.1 ± 0.8
Older adolescents (n = 412)	12.1 ± 5.4	2.2 ± 0.5
		
t	10.7995	20.555
p value	<0.0001	<0.0001
**Class level**		
SS1 (n = 347)	7.3 ± 2.2	2.4 ± 0.3
SS2 (n = 691)	9.7 ± 3.8	2.4 ± 0.4
SS3 (n = 62)	12.6 ± 4.5	1.8 ± 0.2
		
F	90.8591	80.2367
p values	<0.0001	0.003

SS = Senior Secondary.

From the worst responses provided on the CDI by each of the participants, we observed that "most days I do not feel like eating" was the most prevalent (38.6%) response reported by the participants. This was followed by "things bother me all the time" (26%) and the least was "I do everything wrong" (2.5%). Suicidal ideation flagged by "I want to kill myself" presented in 101 (9.2%) of the participants having the same proportion with the feelings of "Nobody really loves me" and "All bad things are my fault".

A summary of the frequency of physical activity participation by the adolescents at different times in the last seven days of the week shows that 27.2% (from private schools) and 14.8% (from public schools) hardly engaged in vigorous activity (such as playing hard, running, jumping and throwing) during their physical education sessions. Besides eating during lunch, 32.1% and 28.6% of the adolescents in the private and public schools respectively reported sitting down (talking, reading and doing school work) in the last seven days. Only 37.3% and 44.2% of the adolescents in the private and public schools claimed they quite often (about 5-6 times in the last week) did physical things in their free time.

### The relationship between depression and physical activity

Using the Pearson's product moment correlation test on the data shows a significant inverse relationship (r = -0.82, p < 0.001) between the CDI and PAQ-A scores. The high correlation obtained between these two variables further produced a coefficient of determination of 0.67. This implies that 67% of the total variation in depression of the participants may be explained by the linear relationship between depression and physical activity.

### Individual and school factors associated with depression and low physical activity among the adolescents

Having identified that there were some adolescents with depressive symptoms and low physical activities, we further carried out bivariate logistic regressions to determine the factors that were significantly associated with these two problems. Two regression analyses were carried out. The first for all the adolescents with mild/moderate and definite depressive symptoms constituting 29.4% of the participants and the second for those with low physical activities (53.8%). The bivariate analysis shows that all individual and school factors were significantly associated with depression and low physical activity after adjusting for age and/or sex (table 4). The odds of having depressive symptoms was reduced by more than half (OR = 0.42, 95% CI = 0.29-0.71) in adolescents who were moderately active when adjusted for age and sex. Adjusted OR also showed a higher risk of having depressive symptoms in the older adolescents than the younger adolescents (OR = 2.16, 95% CI = 1.81-3.44) and similarly, the older adolescents had almost double the possibility of having low physical activity than the younger adolescents (OR = 1.72, 95% CI = 1.29-2.36). The female participants had about three times more possibility of having depressive symptoms (OR = 2.92, 95% CI = 1.82-3.54) and low physical activities (OR = 2.91, 95% CI = 1.51-4.26) than the males. Being in a private school and in the topmost class in the secondary school increased the risk of depression and low physical activity when adjusted for age and sex. The final model for each of depression and low physical activity combined all the factors that were statistically significant at individual and school levels (table 5). In this final multivariable model, it was observed that all the factors significant at individual and school levels for depression remained significant at combined level except for type of school that lost its significance (OR = 0.86, 95% CI = 0.58-1.76). Also in the model, a combination of both individual and school factors further reduced the odds of having depression in the association between depression and moderate physical activity (individual level) and increased the strength (OR = 4.17, 95% CI = 3.70-4.91) of the association between depression and being in SS3 (school level).

**Table 4:** Bivariate analysis for odds of depression and low physical activity

	OR (95% CI) Depressive symptoms	OR (95% CI) Low physical activity
**Physical activity**†		
Low	1	
Moderate	0.42 (0.29-0.71)	
High	0.89 (0.77-1.50)	
		
**Adolescence**††		
Young adolescent	1	1
Older adolescent	2.16 (1.81-3.44)	1.72 (1.29-2.36)
		
**Sex**§		
Males	1	1
Females	2.92 (1.82-3.54)	2.91 (1.51-4.26)
		
**Type of school**†		
Public	1	1
Private	1.73 (1.56-2.58)	0.77 (0.47-0.86)
		
**Class of student**†		
SS1	1	1
SS2	1.54 (0.63-2.17)	1.72 (0.98-2.15)
SS3	3.4 (2.55-4.37)	4.79 (3.88-5.61)

SS = Senior Secondary.

† Adjusted for age and sex.

††Adjusted for sex.

§ Adjusted for age.

**Table 5:** Multivariate analysis showing risks of depression and low physical activity by individual factors, school factors and both individual and school factors

	Risk of depression			Risk of low physical activity		
	OR (95% CI) Individual factors	OR (95% CI) School factors	OR (95% CI) Individual and school factors	OR (95% CI) Individual factors	OR (95% CI) School factors	OR (95% CI) Individual and school factors
**Individual Level**						
**Physical activity**						
Moderate	0.66 (0.42-0.77)		0.44 (0.29-0.75)			
High	0.73 (0.61-0.96)		0.74 (0.60-0.95)			
						
**Level of adolescence**						
Older adolescent	1.79 (1.36-2.48)		1.75 (1.28-2.55)	2.61 (1.58-3.72)		2.99 (1.48-4.74)
						
**Sex**						
Females	2.81 (1.92-3.84)		2.82 (2.00-3.79)	3.13 (2.00-5.66)		4.35 (2.79-4.87)
						
**School level**						
**Type of school**						
Private		1.60 (1.18-2.24)	0.86 (0.58-1.76)		1.86 (1.26-2.81)	2.65 (1.52-4.22)
						
**Class of student**						
SS3		3.65 (2.44-4.96)	4.17 (3.70-4.91)		2.19 (1.39-4.10)	2.86 (1.38-4.06)

## Discussion

The main findings from this study were (1) about one fifth of all the adolescents reported symptoms of mild to moderate depression while more than half of them reported low physical activity levels (2) there was a significant inverse relationship between the depression scores of the adolescents and their physical activity scores with moderate physical activity being linked with lower risk of depression (3) Both individual and school factors were associated with depression and low physical activity, with being an older adolescent, female and in the most senior secondary class having significant links with depression and low physical activity among the adolescents. It was also found that compared with the public schools, physical activity was significantly lower and depression was significantly higher in the adolescents attending the private schools.

Data on the precise prevalence and level of depression among adolescents in Nigeria appear to be quite scant, but the prevalence of students experiencing severe depressive symptoms in this study (5.7%) is quite similar to that reported by a few other studies. Adewuya et al [18] reported a prevalence of major depressive disorder of 6.9% among a group of Nigerian adolescents with females having significantly higher prevalence than males. In a study to examine the proportion of children with psychiatric disorders attending primary care in a Nigerian setting, Gureje et al [19] also reported that depressive disorders were present in 6.0%, anxiety-related disorders in 4.7%, and conduct disorders in 6.1% of the children.

The present study found that more than half of the participants actually presented with low physical activity levels indicating that the adolescents were not engaging in sufficient physical activity that could benefit their mental health status. According to the Australian Government Department of Health and Ageing [20], adolescents between 12 and 18 years old should engage in at least 60 minutes of moderate to vigorous physical activity every day to keep healthy. However, where children have been inactive, 30 minutes of moderate activity per day is recommended and should be built up gradually. A study by Nikapota [21] reported that developing countries are subject to rapid socio-cultural and political changes which affect the life-styles of children and their families and hence their physical and emotional well-being. The present study implies that the sampled Nigerian adolescents were not sufficiently active. The high prevalence of low physical activity as well as the prevalence of depression seen in the sample may be indicative of a link between depression and physical activity. A previous study [22] describing physical exercise as a means of being physically active, had documented the link between physical exercise and depression by reporting that exercise withdrawal actually resulted in increased depressive symptomatology in healthy, non-depressed individuals.

The female adolescents in this study had higher depression scores and lower physical activity scores compared to the males. There may be many reasons for this however, it may also be an indication of the link between low physical activity and depression since the female participants in this study had shown a lower level of physical activity. For instance, group-based physical exercise programmes, which can increase daily physical activity or social relationships, have been observed to improve not only physiological fitness levels but also the depressive state and psychophysical stress conditions of participants [8]. Berlin et al [22] also found that depressive symptomatology was more prevalent among sedentary than physically active individuals. The fact that the female adolescents in our sample had a higher depression score is well-recognized and confirms the reports of previous studies. The female adolescents had close to three times higher risk of having depressive symptoms than the males. In the study by Adewuya et al [18] on Nigerian adolescents, the females were also observed to have higher prevalence of depression than the boys, but the authors claimed that there was no age-gender interaction in the findings. It was however reported in a previous study that more boys participated in physical activity than girls, and probably as a result of the link between physical activity and depression, more of the girls than boys reported feelings of sadness, including considering and planning suicide [23].

Higher scores of depression and lower scores of physical activity were seen in the older adolescents compared to the younger ones and among those in the higher classes of study compared to those in the lower classes. Expectedly, the students' ages increase as their class of study increases and a higher class of study implies heavier workload. A combination of heavier workload and anxiety trailing the anticipation of the forthcoming final examinations at the highest level of secondary education could have placed the participants on a tighter academic schedule making it difficult for them to engage in purposeful physical activities. The same situation could also have influenced their psychological state making them present with higher depression scores. It was also observed that older adolescents had higher risk of having depressive symptoms compared to the younger adolescents while being in the topmost class more than tripled the risk of having depressive symptoms and increased more than four folds the risk of having low physical activity. This may be because the highest class of study (SS3) was occupied mainly by the older adolescents who happened to have higher risk of depression and low physical activities.

About one third of the adolescents in both the private and public schools were found to be sedentary for most part of the day while suicidal ideation previously reported to be about 20% and above in studies by Omigbodun et al [24] and Daley et al [25] was about 9% in this study. It is however important to note that the difference in the prevalence of suicidal ideation in this report and that of Omigbodun et al [24] which also surveyed a group of Nigerian adolescents may be due to a number of reasons. First, Omigbodun et al [24] measured suicidal behaviour using the Diagnostic Interview Schedule for Children (DISC) (Predictive Scales 432 - items 23-25) while the observation in this present study was a response to one of the questions on the CDI. Second, they conducted their study on both urban and rural adolescents while this study was limited to urban adolescents. Third, they considered all grades in the school while only the senior grade was considered in this study. Multiple psychosocial factors such as sexual abuse, physical attack and involvement in physical fights were found to be the significant predictors of suicidal behaviour among Nigerian adolescents as reported by Omigbodun et al [24]. Despite the lower prevalence of suicidal ideation noted in this study, this is an area worthy of further research investigation. Depression has been reported to be the most important predictor of suicide, and failure to address depression in adolescents can lead to an increase in cases of suicides [26].

The coefficient of determination shows that a substantial variation in depression in adolescents may be explained by physical activity. Because the relationship may not be causal, the remaining variation seen in the depression of the adolescents may be explained by other factors that were not considered in the study. These factors may include the socioeconomic status of the parents and the presence of co-morbidities. An earlier cross-sectional analysis however, reported an association between physical activity and depression even when adjustments were made for a relatively large number of potentially confounding variables [7]. This association may be because of the likely link between physical activity and depression. According to Rothon et al [10], no clear mechanism for the association between physical activity and depression has been established, but biochemical, physiological and psychological mechanisms have been proposed. The authors claimed that one of the explanations relates to the indirect effect that physical activity has on mood through providing increased opportunities for social interaction. It will be appropriate to state here however, that the association existing between physical activity and depression may actually be bidirectional. As presented in this study that depression is linked with low physical activity, it is also plausible for low physical activity to be linked to increasing depression. In a comprehensive review of published studies on correlates of physical activity in children and adolescents, a high level of depression was consistently associated with low physical activity in adolescents [27]. This study is not able to say however whether depression precedes physical inactivity or physical inactivity precedes depression.

This study showed that adolescents with moderate physical activity had a reduced risk of having depression after adjustment for age and sex. In a previous study, it was also indicated that low to moderate intensity physical activity was a protective factor against depression and psychotic symptoms in Chinese adolescents [6]. However, it was found in the study that high-intensity physical activity was not a protective factor against psychological disorders, but rather a risk factor for general mental health problems and hostility. In this present study, high physical activity reduced odds of having more severe depressive symptoms with a trend level finding (non significant) that high physical activity was linked with reduced risk of depression.

This study further found that the adolescents in private schools had a higher risk of low physical activity than those in public schools. No previous reports were found on the variation of physical activity between the students of private and public schools, but it is assumed that this disparity may have a lot to do with the socioeconomic background of the students. It is possible that the students in the public schools, hypothetically from low socioeconomic backgrounds had to exert more physical effort in executing their daily routines. This may include trekking to school and the use of manual force for their daily chores contrary to the life of the adolescents from higher socioeconomic backgrounds that abound in the private schools. This may explain the link between low physical activity and higher depression as seen in the students of private schools.

This study should be interpreted within the confines of its scope, limitations and strengths. The fact that this study was cross-sectional prevents any inference of causality. The school-based nature of the study also means that findings cannot be generalised to adolescents who do not attend school. However, apart from providing an organised research site, the schools were also considered as an avenue where the issues of depression and physical activity among adolescents could be effectively championed. According to Grzywacz and Fuqua [28] schools are in a position to prevent public health concerns such as depression. Our mode of assessment using the questionnaires may not be the most precise method because of the possibility of substantial recall bias associated with self reports; however, questionnaires have produced reliable assessments of numerous constructs. According to Corder et al [29], self-report methods may still be the only feasible way to assess physical activity in many situations and are important for assessing aspects of physical activity not easily measured objectively, such as mode and domain.

The relationships found in this study are not necessarily causal because there may be other potential confounders of depression apart from age and sex that this study did not investigate. Such confounders include health status, self esteem and the socioeconomic status of parents. There is also the possibility of residual confounding in this study which may be due to possible flaws in our assessment or the fact that we did not measure some other confounders outrightly. Further research may need to look at how issues such as levels of school and family care, motivation and serious life events will interact with depression and physical activity of the adolescents. However, all our analyses pointed to the fact that those who had lower physical activities had higher levels of depressive symptoms. This study identifies that there is a need to further explore the complex link between physical activity and depression among Nigerian adolescents, just like their counterparts in other developing countries. Furthermore, this study will provide an additional basis for exploring physical activity as complementary therapy in the intervention for depression among adolescents given its low-cost and the increasing cost of mental health care. Also from the outcome of this study, there is an urgent implication for government policy decision reviews based on the fact that there is a large number of adolescents who are not physically active and due to the fact that close to 6% of the students were experiencing quite severe depressive symptomatology.

In conclusion, the results of this study showed a sizable burden of both mild to moderate and definite symptoms of depression in addition to a prevalent level of low physical activity among the adolescents. There was an inverse relationship between depression and physical activity and both individual and school factors were linked with depression and low physical activity. Being an older adolescent, a female and in the most senior secondary class were the main contributors to both depression and low physical activity among the adolescents. For future research, we suggest longitudinal studies to shed light on causal issues and studies that will examine the possible effects of physical activity among clinical samples of adolescents with depression.

## Competing interests

The authors declare that they have no competing interests.

## Authors' contributions

AFA was involved in the conceptualization, design, statistical analysis, interpretation of data, editing for intellectual content and manuscript preparation. NCO was involved in the conceptualization, data collection, literature search and manuscript preparation. CYA was involved in the conceptualization, editing for intellectual content and manuscript preparation. All the authors read and approved the final manuscript.

## Authors Information

AFA is a lecturer in the Department of Physiotherapy, University of Ibadan and Honorary Clinical Consultant in Physiotherapy, University College Hospital, Ibadan, Oyo State, Nigeria. NCO is a graduate physiotherapist of the Department of Physiotherapy, College of Medicine, University of Ibadan, Ibadan, Nigeria. CYA is a senior registrar in the Department of Psychiatry, University College Hospital, Ibadan, Nigeria.
